# Genetic and regulatory mechanism of susceptibility to high-hyperdiploid acute lymphoblastic leukaemia at 10q21.2

**DOI:** 10.1038/ncomms14616

**Published:** 2017-03-03

**Authors:** James B. Studd, Jayaram Vijayakrishnan, Minjun Yang, Gabriele Migliorini, Kajsa Paulsson, Richard S. Houlston

**Affiliations:** 1 Division of Genetics and Epidemiology, The Institute of Cancer Research, 15 Cotswold Road, Sutton, London SM2 5NG, UK; 2 Department of Laboratory Medicine, Division of Clinical Genetics, Lund University, BMC C13, Lund SE-221 84, Sweden; 3 Division of Molecular Pathology, The Institute of Cancer Research, 15 Cotswold Road, Sutton, London SM2 5NG, UK

## Abstract

Despite high-hyperdiploid acute lymphoblastic leukaemia (HD-ALL) being the most common subgroup of paediatric ALL, its aetiology remains unknown. Genome-wide association studies have demonstrated association at 10q21.2. Here, we sought to determine how this region influences HD-ALL risk. We impute genotypes across the locus, finding the single nucleotide polymorphism rs7090445 highly associated with HD-ALL (*P*=1.54 × 10^−38^), and residing in a predicted enhancer element. We show this region physically interacts with the transcription start site of *ARID5B*, that alleles of rs7090445 have differential enhancer activity and influence *RUNX3* binding. *RUNX3* knock-down reduces *ARID5B* expression and rs7090445 enhancer activity. Individuals carrying the rs7090445-C risk allele also have reduced *ARID5B* expression. Finally, the rs7090445-C risk allele is preferentially retained in HD-ALL blasts consistent with inherited genetic variation contributing to arrest of normal lymphocyte development, facilitating leukaemic clonal expansion. These data provide evidence for a biological mechanism underlying hereditary risk of HD-ALL at 10q21.2.

## Abstract (web summary)

Risk for the paediatric cancer high-hyperdiploid acute lymphoblastic leukaemia (HD-ALL) has been associated with genetic variants at 10q21.2. Here, the authors characterize this region, establishing a single risk variant and showing its role in dysregulated expression of *ARID5B*.

Acute lymphoblastic leukaemia (ALL) is the most common childhood malignancy. Twenty to twenty-five per cent of ALL is characterized by high-hyperdiploidy (51–67 chromosomes), making high-hyperdiploid ALL (HD-ALL) one of the major subgroups of paediatric cancer^1^. A characteristic genetic feature of HD-ALL is the non-random gain of chromosomes X, 4, 6, 10, 14, 17, 18 and 21, with individual trisomies or tetrasomies seen in over 75% of cases^2^. High-hyperdiploidy is almost exclusively observed in the context of paediatric precursor B-cell ALL, with a peak incidence at 4 years of age^2^, and has a more favourable outcome than other forms of B-cell ALL (ref. 3). The aetiology of HD-ALL remains unknown, however several lines of evidence are consistent with the initiating transforming event occurring *in utero*^4^,^5^,^6^, suggesting additional secondary events are required to trigger clonal expansion and the development of overt disease.

Subsequent clonal expansion is likely to be influenced by both inherited genetic susceptibility, and as yet unidentified environmental risk factors. A number of genetic disorders are associated with an increased risk of ALL including Down’s syndrome^7^ and ataxia telangiectasia^8^. However, even collectively these account for only a small proportion of ALL (ref. 9) and analyses of risk in siblings of cases suggest that common genetic variation contributes to disease susceptibility^10^.

Recently, independent genome-wide association studies (GWAS) demonstrated robust association at a locus in the gene AT rich interactive domain 5B (*ARID5B*) at 10q21.2 (refs 11, 12). Variation at this locus is primarily associated with HD-ALL (refs 11, 12), having no impact on the risk of *ETV6/RUNX1* translocation positive ALL. Currently it is unclear how 10q21.2 influences the risk of developing HD-ALL. Elucidating the function of this risk locus is therefore an important step towards the development of testable hypotheses regarding the biological processes involved in the pathogenesis of HD-ALL.

Here, we sought to identify the causal polymorphism(s) driving the 10q21.2 genetic association with ALL susceptibility as a basis for understanding HD-ALL initiation and addiction mechanisms. We identify a potential mechanism contributing to the additional risk to ALL conferred by 10q21.2. Variation at rs7090445 disrupts *RUNX3* binding and via a looping interaction reduces the expression of *ARID5B*. Consistent with rs7090445 contributing to ALL leukaemic blasts with the C-risk allele have reduced *ARID5B* expression and preferentially duplicate the copy of chromosome 10 harbouring this variant.

## Results

### Fine mapping and epigenomic profiling of the 10q21.2 locus

**Figure 1:**
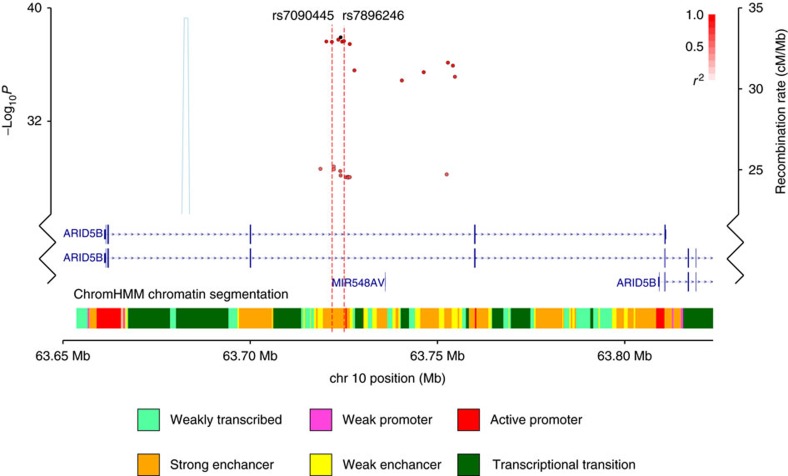
Genetic mapping and overall epigenetic landscape of the 10q21 HD-ALL risk locus. The region of association maps to an 8 kb haplotype block within intron 3 of *ARID5B*. SNPs (dots) are shown based on their chromosomal position (GRCh37/hg19 human genome build) on the *x* axis and −log_10_ association *P*-value in HD-ALL on the y axis. Colour intensity of each SNP reflects the extent of linkage disequilibrium (white *r*^2^=0 to dark red *r*^2^=1) with the lead SNP, rs10821936 (shown in black). Recombination rates, estimated using 1,000 genomes and UK10K samples of European ancestry, are shown by a light blue line. Gene positions and chromatin state segmentation (ChromHMM) in GM12878 from ENCODE project data are shown. Also annotated are the candidate functional SNPs rs7090445 and rs7896246, red dotted lines.

**Figure 2:**
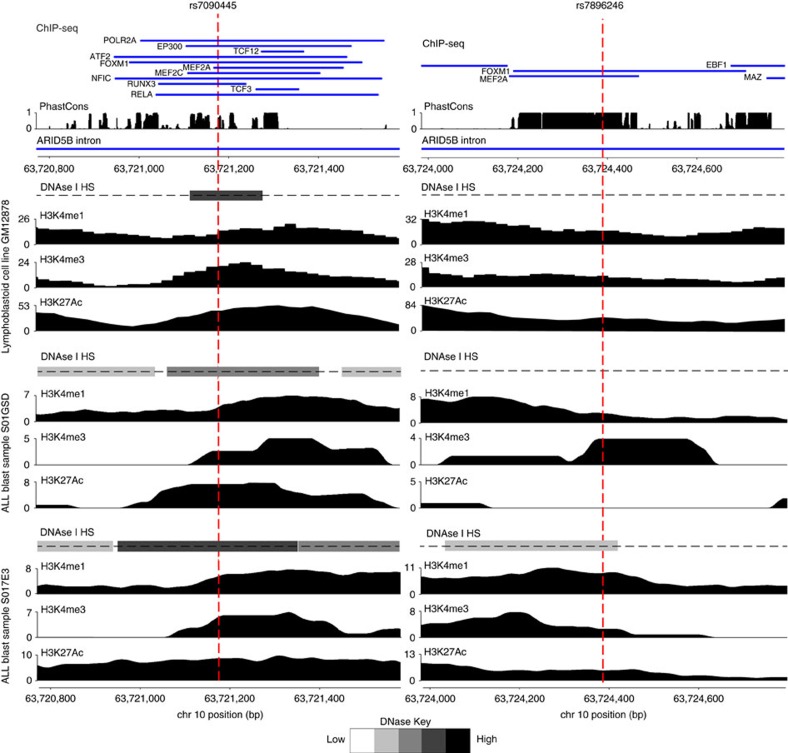
Epigenetic profiles surrounding the 10q21 SNPs rs7090445 and rs7896346. Transcription factor ChIP-seq from GM12878 (blue bars) is shown in the upper panel, annotated with the ChIP’d TF name. Evolutionary conservation quantified by PhastCons score, shown below. ChIP-seq data for H3K4Me1, H3K4Me3 and H3K27Ac histone modifications and DNaseI hypersensitivity (DNASe I HS) for the lymphoblastoid cell line GM12878 from ENCODE and for ALL blasts from the Blueprint Project. Extent of DNaseI hypersensitivity based on normalized DNAse-seq data from respective cells. Base pair positions are from GRCh37/Hg19. Figures were generated in visPIG (ref. 37).

We first fine mapped the 10q21.2 risk locus by imputation using UK10K and 1000 Genomes Project as reference and data from two GWAS data sets totalling 465 HD-ALL cases, both previously reported^13^. Supplementary Table 7 details whether SNPs in the 10q21 risk loci were directly genotyped or imputed. This identified eight SNPs with minor allele frequency (MAF) >0.01, an association *P*<1.0 × 10^−35^ (fixed-effects meta-analysis of logistic regression *P* value) and an odds ratio of >2.4 at 10q21.2 in HD-ALL (Fig. 1, Supplementary Table 8). All eight SNPs localize to intron three of *ARID5B* and are in strong linkage disequilibrium (LD) defining a single risk haplotype (LD to the lead SNP rs10821936 *r*^2^=0.88–1.00; Supplementary Table 8 and Supplementary Fig. 1). We further prioritized associated variants by assessing their regulatory potential inferred through B-cell specific DNase I hypersensitivity mapping and chromatin immunoprecipitation sequencing (ChIP-seq) experiments in the lymphoblastoid cell line (LCL) GM12878 and ALL blasts from the ENCODE^14^ and Blueprint^15^ projects respectively (Fig. 2, Supplementary Tables 9 and 10). These data highlighted SNPs rs7896246 and rs7090445 (logistic regression *P* values 1.36 × 10^−38^ and 1.54 × 10^−38^, respectively) as plausible functional SNPs based on their putative enhancer characteristics, defined by relevant histone markers (H3K27ac, H3K4me1 and H3K4me3), transcription factor binding, DNase I hypersensitivity and sequence conservation (Fig. 2; Supplementary Fig. 1).

### rs7090445, rs7896246 and *ARID5B* expression

**Figure 3:**
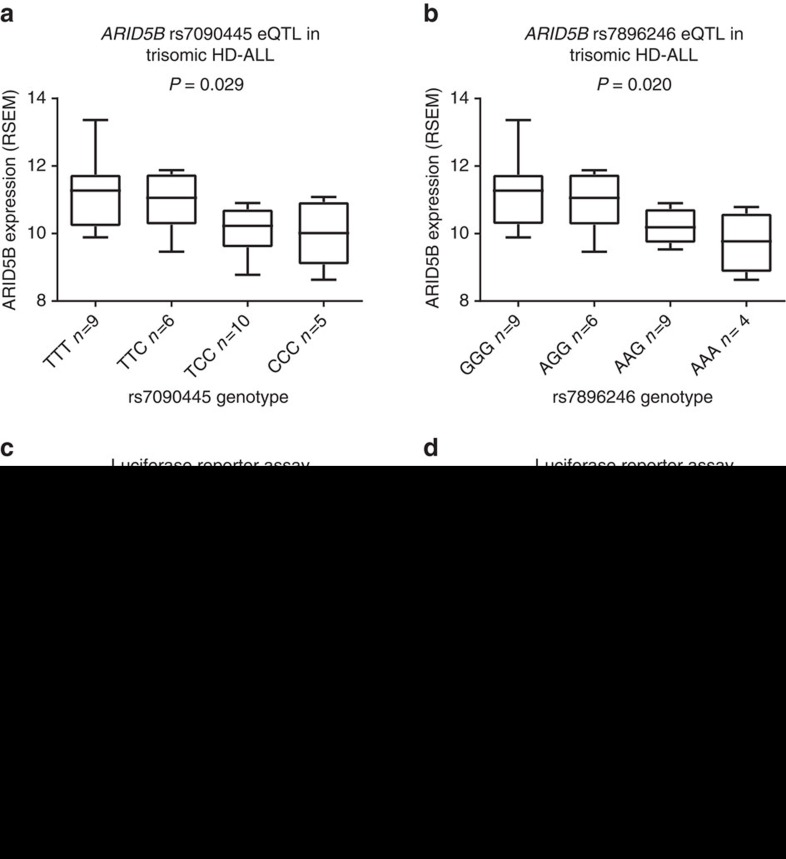
rs7090445 is associated with lower *ARID5B* expression and reduced enhancer activity. Expression quantitative trait loci (eQTL) analysis performed in HD-ALL trisomic for chromosome 10 for (**a**) rs7090445 and (**b**) rs7896246. Difference in RNA sequencing of *ARID5B* expression (RSEM) assessed by ANOVA test. rs7090445-C and rs7896246-A risk alleles show decreased expression over the protective allele. Allele-specific constructs containing the 818bp putative regulatory sequence flanking rs7090445 were cloned into the pGL3-promoter luciferase reporter vector and transfected into (**c**) REH and (**d**) SEM cell lines. The ratio of luminescence from the experimental pGL3-rs7090445 constructs to the Renilla internal control, pRL-SV40, normalized to the empty pGL3-SV40 promoter vector. Data shown are mean±s.e.m. from six independent experiments performed in triplicate. Difference in gene expression assessed by Student’s *T*-test. Luciferase data were normally distributed. The rs7090445-C risk allele has significantly decreased enhancer activity over the protective allele.

We next examined if the genotypes of rs7090445 or rs7896246 were associated with *ARID5B* expression. Gene expression was quantified from RNA-sequencing data in 45 HD-ALL cases, of which the majority, 30 (65%), were trisomic for chromosome 10, consistent with previous findings^2^. To control for gene dosage we restricted our expression quantitative trait locus (eQTL) analysis to these trisomic blasts. Genotypes of both rs7090445 and rs7896246 were associated with *ARID5B* expression, with the rs7090445-C and rs7896246-A risk alleles associated with reduced expression (ANOVA *P*=0.029 and *P*=0.020, respectively, Fig. 3a,b). The risk alleles of rs7090445 and rs7896246 were also associated with reduced *ARID5B* expression in disomic cases, albeit non-significantly (Supplementary Fig. 2). Following this we tested for an eQTL with *RTKN2* and *C10orf107* which map within a 500 kb window of rs7090445 and rs7896246. No eQTL was detected for *RTKN2* (*P*>0.05) (Supplementary Fig. 3), and C10orf107 was not expressed.

### rs7090445 genotype influences enhancer activity

To measure the effect of rs7090445 and rs7896246 alleles on enhancer activity we performed luciferase reporter assays in MHH-CALL2 and SEM BCP ALL cell lines. Transfection with constructs containing the rs7090445 risk C-risk allele displayed significantly lower normalized luminescence compared to non-risk T-allele constructs (Two-tail *t*-test *P*=0.008 and *P*=0.028, Fig. 3c,d, respectively). rs7896246 genotype did not impact on enhancer activity (Supplementary Fig. 4a,b). These data are thus consistent with a model of HD-ALL risk in which variation at rs7090445 is associated with decreased expression of *ARID5B*.

### rs7090445 modulates RUNX3 binding affinity

**Figure 4:**
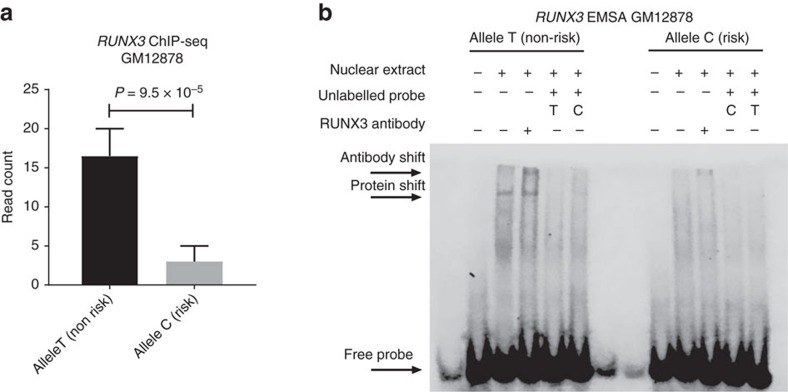
The rs7090445 risk allele disrupts RUNX3 binding. (**a**) ENCODE ChIP-seq data from the rs7090445 heterozygote lymphoblastoid cell line GM12878. Mean read counts for each allele were combined from replicates, error bars show SEM. Meta *P* value calculated using Fisher’s method combining binomial test *P* values. (**b**) Electromobility shift assay (EMSA) in GM12878 showing differential nuclear protein binding to alleles of rs7090445. Increased protein binding observed for the non-risk T allele was shifted by the addition of a RUNX3 antibody. An uncropped EMSA gel image is provided in Supplementary Fig. 9.

We next interrogated GM12878 Encode ChIP-seq data to identify differential transcription factor (TF) binding for alleles of rs7896246 and rs7090445. Reads mapping to each SNP allele from experiments were mapped and enumerated (Supplementary Table 9). Only *RUNX3* at rs7090445 displayed a significant read bias, with a 1.7-fold excess of reads mapping to the non-risk T allele (Weighted Fisher’s combined binomial *P*<0.001, Fig. 4a). To exclude the possibility this observation was a consequence of copy number or other local *cis* variation we analysed whole genome sequencing data from the same cell line finding no evidence of allelic read bias (Binomial *P*=1.0) or any other genetic variation 250 bps upstream or downstream of rs7090445, sequencing coverage within this interval was at least 45X. To verify that *RUNX3* also binds to rs7090445 in MHH-CALL2 we performed ChIP-qPCR; enrichment for rs7090445 was observed relative to IgG isotype controls, consistent with differential *RUNX3* binding driving allele specific enhancer activity (Supplementary Fig. 5). We examined the possibility that variation at rs7090445 disrupts overlapping motifs using *in silico* data from HaploReg v4.1 (ref. 16) in conjunction with ENCODE data. While motifs for CUX1, HOXD8 and MEF2A were possibly disrupted by variation at rs7090445 the surrounding loci contains mismatches at high stringency bases for each TF and ENCODE data provided no evidence for CUX1 or MEF2A binding (Supplementary Table 9; Supplementary Fig. 6). To further examine the influence of rs7090445 genotype on function we performed an EMSA in GM12878. Nuclear protein bound to the non-risk T allele of rs7090445 with greater affinity than the risk C allele. The addition of an anti-*RUNX3* antibody resulted in a supershift suggesting *RUNX3* is a component of this complex (Fig. 4b). Hence while rs7090445 does not overlap with a predicted *RUNX3* binding motif, this is a common feature of ChIP-seq data, and suggests *RUNX3* may bind cooperatively with as yet unidentified additional factors^17^,^18^,^19^.

### rs7090445 risk region interacts with the *ARID5B* promoter

**Figure 5:**
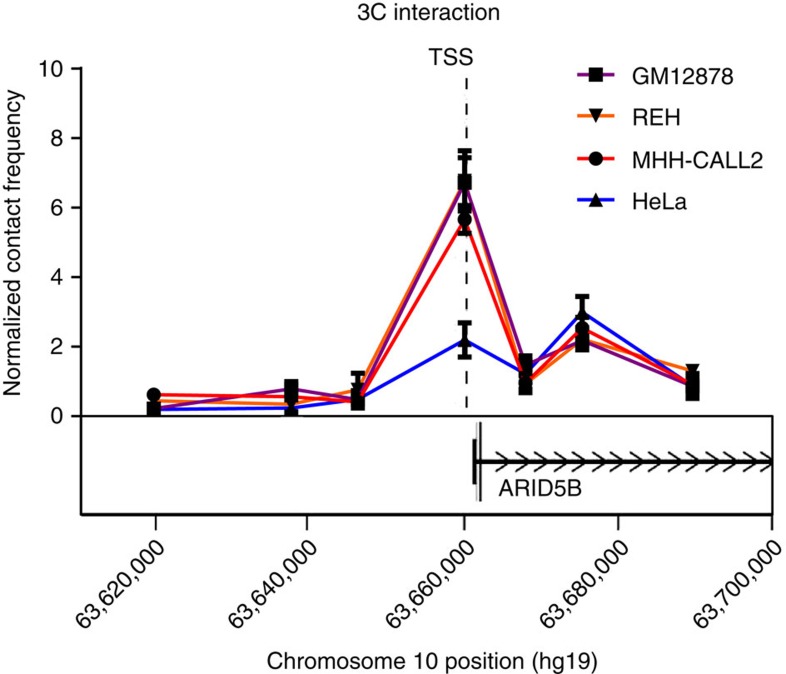
3C interaction plot of rs7090445 with the *ARID5B* promoter. Physical map of 10q21 showing the relative interaction frequencies calculated from the abundance of ligation products formed between the constant fragment, containing rs7090445, and each of the target fragments (*ARID5B* promoter fragment or surrounding genomic intervals). Data shows mean qPCR abundance ±s.e.m., normalized to the abundance of inter-site control region. The assay was performed independently four times for GM12878, REH and HeLa, and three times for MHH-CALL2. GM12878, MHH-CALL2 and REH show a significant increase in the contact frequency between rs7090445 and *ARID5B* TSS compared to both HeLa and cell specific back ground levels, Students *t*-test *P*<0.05 two sided.

Various lines of evidence indicate that chromatin looping interactions formed between enhancer elements and gene promoters map within distinct chromosomal topological associating domains (TADs). To examine the TAD structure of the 10q21 rs7090445 region and identify local chromatin patterns, we analysed publicly accessible Hi-C GM12878 data^20^ as a source of B-cell information (Supplementary Fig. 7a). These data demonstrated the transcription start site (TSS) of *ARID5B* and the rs7090445 risk region map within a single TAD (Supplementary Fig. 7a). Furthermore, accepting the caveat of 5 kb resolution of the Hi-C data, within this TAD the most highly enriched contact of the rs7090445 risk region is with the TSS of *ARID5B*, which showed a 2.7-fold enrichment over expected levels (Supplementary Fig. 7b), consistent with a looping interaction between these regulatory elements. Following from this observation we performed 3C-qPCR in REH and MHH-CALL2 ALL cell lines, GM12878 and HeLa cells to determine chromatin looping interaction between the genomic region encompassing rs7090445 and the *ARID5B* promoter. From 3C-qPCR we demonstrated that rs7090445 forms chromatin looping interaction with the TSS of *ARID5B* in cells of B-cell lineage but not in HeLa cells (Fig. 5). Collectively these data suggest a mechanism by which *RUNX3*-bound rs7090445 regulates *ARID5B* expression in a tissue-specific manner. The genotype of rs7090445 did not affect the contract frequency between these two loci (Supplementary Table 11).

### *RUNX3* regulates *ARID5B* expression

**Figure 6:**
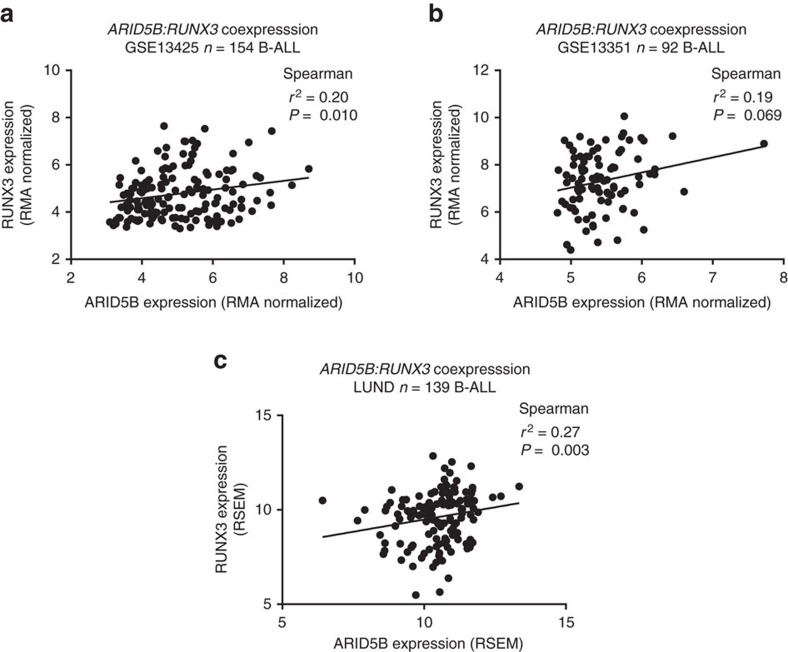
*RUNX3* and *ARID5B* expression are correlated in B-cell precursor ALL. Expression of *ARID5B* and *RUNX3* was examined in the BCP-ALL expression data sets (**a**) GSE13425, (**b**) GSE13351 and (**c**) LUND (ref. 22). Lines show linear regression fits. Expression correlation was assessed by Spearman’s *r*^2^ and *P* value test. Combined *RUNX3:ARID5B* correlation *P* value 5.6 × 10^−5^ (weighted Fisher’s method).

**Figure 7:**
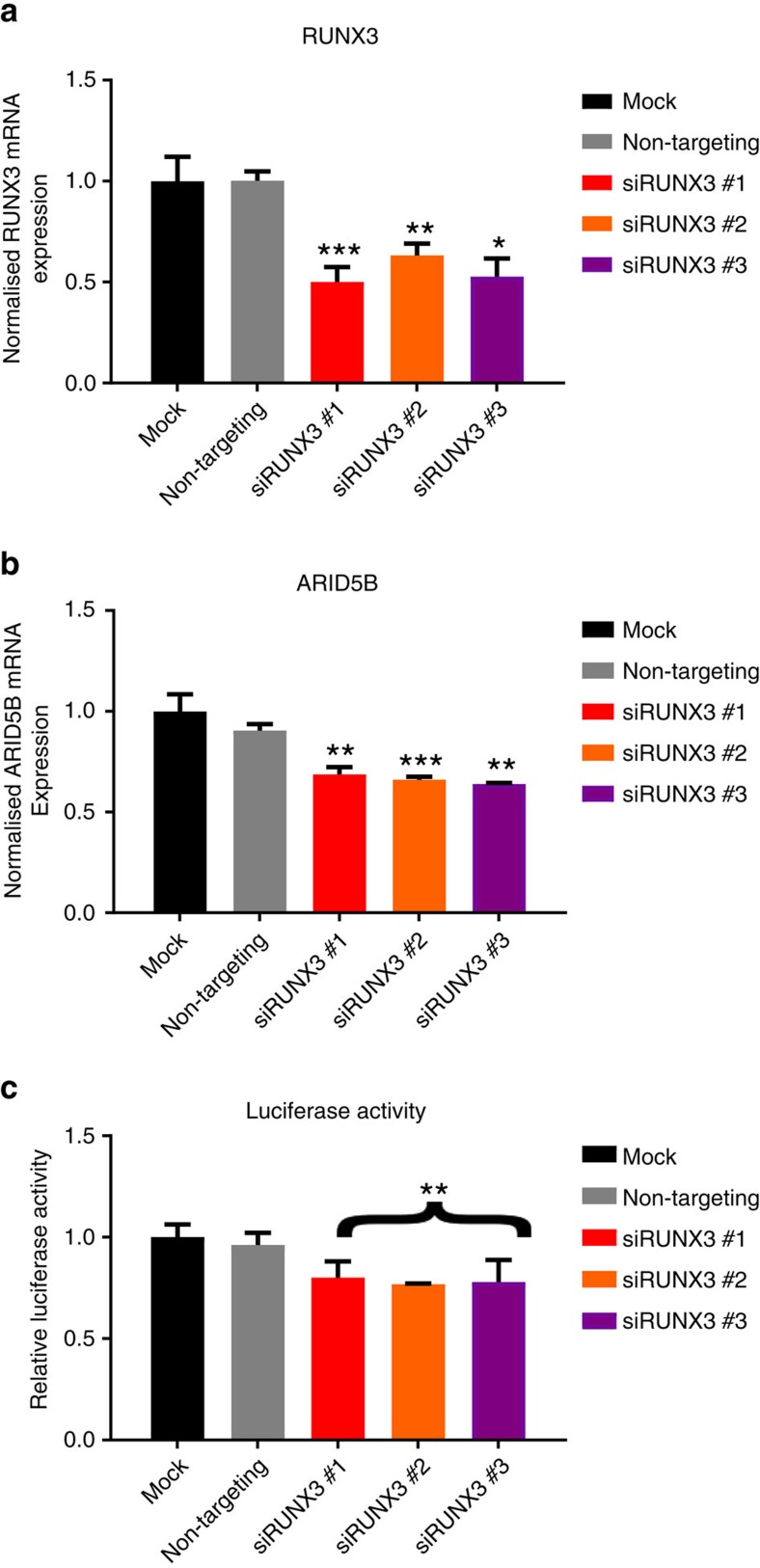
*RUNX3* drives *ARID5B* expression and enhancer activity of rs7090445. mRNA expression quantitation of siRNA knockdown in the lymphoblastoid cell line GM11832. Cells were transfected with one of three *RUNX3* siRNAs for 48 h, after which cells were assayed for expression of *RUNX3* (**a**) *ARID5B* (**b**) and re-transfected with the high activity T allele of the rs7090445 pGL3 Promoter reporter construct (**c**). Gene expression data in **a**,**b** normalized to the geometric mean of PPIA, TBP, G6PD and LAMIN A/C. Data shown are mean ±s.e.m. from *n*=4 (**a**,**b**), *n*=3 (**c**). Asterisk show the *P* value of a two sided Students *t*-test (*P<0.05, **P<0.01, ***P<0.001). qRT-PCR expression data were normally distributed.

To support a role for *RUNX3* in *ARID5B* regulation we analysed tumour gene expression data from three cohorts of paediatric B-ALL totalling 385 cases (GSE13425, GSE13351 (ref. 21) and LUND (ref. 22) data sets). In each series *ARID5B* and *RUNX3* expression were positively correlated (Fig. 6; Weighted Fisher’s combined Spearman’s *P*=5.6 × 10^−5^). While a consistent association was shown between *ARID5B* and *RUNX3* gene expression in HD-ALL the small size of these subsets of the data may have precluded demonstration of a statistically significant relationship. No consistent correlation between *ARID5B* and *RUNX3* was observed in *ETV6/RUNX1* positive ALL (Supplementary Fig. 8). To establish a direct relationship between *RUNX3* and *ARID5B* expression we conducted small interfering RNA (siRNA) experiments. Since transfection efficiency was found to be poor in MHH-CALL2 cells, siRNA *RUNX3* knockdown was performed in GM11832 (homozygous for rs7090445 T). We also confirmed that RUNX3 binds to rs7090445 in GM11832 by ChIP-qRT (Supplementary Fig. 5). Knockdown of *RUNX3* was accompanied by a significant reduction in *ARID5B* messenger RNA (mRNA) (Two-tailed *t*-test *P*=2.4 × 10^−3^, *P*=7.0 × 10^−4^, *P*=1.2 × 10^−3^; *RUNX3* siRNAs 1, 2 and 3, respectively: Fig. 7b). If *RUNX3* contributes to the allele specificity of the rs7090445 reporter assay its depletion should be reflected in reduced luciferase activity. Cells depleted for *RUNX3*, by siRNA, were subsequently transfected with the high activity rs7090445 T-allele pGL3 vector construct. *RUNX3* knockdown was also accompanied by reduced luciferase activity (Fig. 7c).

### rs7090445 risk allele is amplified in hyperdiploid B-ALL

**Figure 8:**
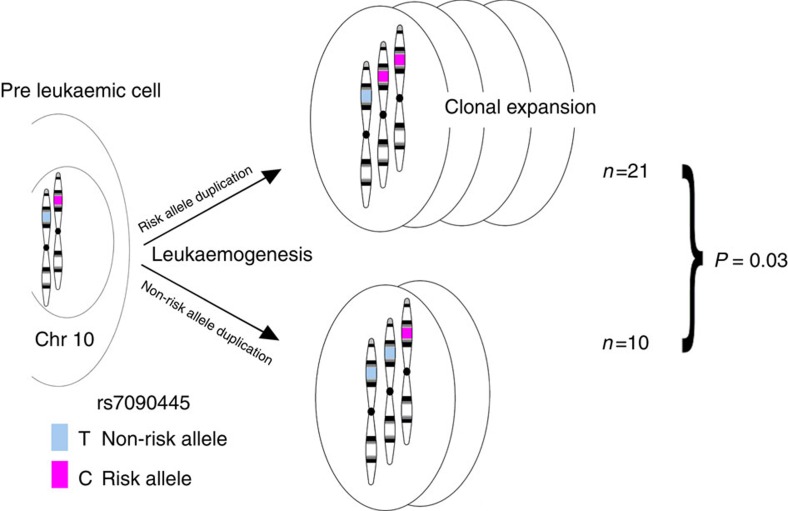
The rs7090445 risk allele is preferentially retained in high-hyperdiploid ALL. The genotype of rs7090445 was interrogated in a series of high-hyperdiploid cases, 31 of which were trisomic for chromosome 10 and heterozygous for rs7090445. *P* value calculated using a one-sided binomial test.

Since, trisomy of chromosome 10 is frequently observed in HD-ALL we sought to determine whether the rs7090445 risk allele, associated with reduced ARID5B expression, is preferentially amplified. To investigate the relationship between heritable risk and somatic mutation in HD-ALL we examined ALL blasts with both chromosome 10 trisomy and heterozygous for rs7090445 (*n*=31). Of these 21 had duplicated the risk allele (C) and 10 had duplicated the non-risk allele (T) (Binomial test *P*=0.035, Fig. 8).

## Discussion

Collectively, our data demonstrate a plausible mechanism underlying the 10q21 risk locus for HD-ALL being mediated through rs7090445, which disrupts *RUNX3* transcription factor binding. Our data are compatible with the rs7090445-C allele conferring increased HD-ALL risk through reduced *RUNX3*-mediated expression of *ARID5B*. CRISPR/Cas9 genome editing of rs7090445 should provide further support for these assertions, given the limitations of luciferase-based assays. Furthermore, epigenetic and chromosome conformation capture data are consistent with rs7090445 localizing within a chromatin contact domain and overlapping a B-cell enhancer. This interval, anchored by CTCF binding sites, forms a ‘loop domain’ which is expected to bring two regions of RUNX3 binding, separated by a linear distance of around 60Kb, into physical contact close to the TSS of *ARID5B*.

Here we have provided direct evidence that *ARID5B* is transcriptionally regulated by *RUNX3*. *ARID5B* plays an important role in embryogenesis and growth regulation. *ARID5B* forms a complex with *PHF2* (ref. 23), which has H3K9me2 histone demethylase activity. H3K9me2 is one of the predominant markers of repressed chromatin and as such *ARID5B* is believed to play a role in epigenetic activation of gene expression. Mouse models with targeted disruption of *ARID5B* have reduced bone marrow cellularity and B220^+^/IgM^−^ B-cell progenitor cells populations^24^. Interestingly ALL blasts lack B220 expression^25^. *RUNX3*, a Runt-related transcription factor has also been implicated in the lineage specification of lymphoid cells where *RUNX3* knock-out are associated with aberrant differentiation of innate lymphocytes^26^.

It has been shown that chromosomal gains in HD-ALL often arise prenatally^4^,^5^,^6^. No recurrent fusion gene-forming rearrangements have however been observed in tumours, and analysis of mutations on trisomic chromosomes indicates that chromosomal gains are early events in tumorigenesis, strengthening the notion that the acquisition of high-hyperdiploidy is the main driver event in this common paediatric malignancy^27^. Moreover sequencing data indicates a latency period after the high-hyperdiploid pattern is established in most patients. Hence the consequence of the chromosomal gains is probably mediated through dosage effects, where additional genetic aberrations are likely to be required for the development of overt leukaemia. We have shown that individuals carrying the rs7090445-C risk allele have reduced *ARID5B* transcript levels, however chromosome 10, containing *ARID5B*, is trisomic in around 70% of HD-ALL. We also show that HD-ALL heterozygotes preferentially duplicate the chromosome 10 homologue carrying the rs7090445-C risk allele, potentially allowing increased dosage of chromosome 10 genes relative to *ARID5B*. The effects of chromosome 10 gain are likely driven by a selective advantage conferred by increased dosage of additional unknown factors unrelated to *ARID5B*. It is therefore possible that preferential gain of the chromosome 10 homologue carrying the risk allele is due to the conflicting selective forces acting to reduce *ARID5B* expression, while simultaneously inducing dosage effects associated with trisomy 10.

In conclusion we have shown that in individuals carrying the rs7090445-C risk allele, *ARID5B* transcript levels are reduced and this provides a mechanistic basis for the 10q21 risk association for HD-ALL. This observation and the finding that the risk allele of rs7090445 is preferentially retained on additional copies of chromosome 10 in HD-ALL blasts are compatible with the hypothesis that inherited genetic variation contributes to decreased *ARID5B* expression and arrest of normal lymphocyte development facilitating leukaemic clonal expansion.

## Methods

### Ethics

Collection of GWAS samples and clinicopathological information from subjects was undertaken with informed consent in accordance with the Declaration of Helsinki and with approval of the ethical review board. HD-ALL blast RNA sequencing and genotyping sample collection was approved by the Ethical Review Board at Lund University, no 2011/289. Consent was obtained from all subjects.

### Genome-wide association study

The United Kingdom (UK)-GWAS and German-GWAS of B-cell precursor (BCP) ALL have been previously reported^11^,^28^. Briefly, the UK GWAS comprised 824 BCP-ALL cases, including 289 with HD-ALL (mean age of diagnosis 5.5 years), genotyped using Illumina Human 317 K arrays (Illumina, San Diego). Controls were provided by the Wellcome Trust Case Control Consortium 2 (http://www.wtccc.org.uk/) of 2,699 individuals in the 1958 British Birth Cohort genotyped using Hap1.2M-Duo Custom array data and 2,501 individuals from the UK Blood Service. The German GWAS comprised 1,155 cases, including 176 with HD-ALL (mean age at diagnosis 6.0 years), genotyped using Illumina Human OmniExpress-12v1.0 arrays. Controls comprised 2,024 healthy individuals from the Heinz Nixdorf Recall study^29^ genotyped using Illumina HumanOmni1-Quadv1 and HumanOmniExpress-12v1.0 arrays. GWAS quality control (QC) has been described previously^28^. To recover untyped genotypes we performed imputation using IMPUTE2 v2.3 (ref. 30) with a combined UK10K (ref. 31) and 1000 Genomes Project (phase III)^32^ panel for reference. Poorly imputed SNPs (INFO score <0.80) were excluded. Frequentist association testing between SNP genotype and HD-ALL was performed using logistic regression under an additive genetic model in SNPTESTv2.5 (ref. 33). Meta-analysis was undertaken under a fixed-effects model using inverse variance weighting in META v1 (ref. 33).

### ENCODE and Blueprint chromatin state dynamics

Epigenetic profiles of association signals are composed of chromatin state segmentation (ChromHMM), DNAse I hypersensitivity, transcription factor ChIP-Seq and histone modification data in the lymphoblastoid cell line GM12878 from the ENCODE project^14^ and in leukaemic blasts from two paediatric precursor B-cell acute lymphoblastic leukaemia cases from the Blueprint project^15^. The individual data sets used are listed in Supplementary Table 10.

### Cell lines

MHH-CALL2, SEM, REH B-ALL and HeLa cell lines were obtained from the DMSZ (Braunschweig, Germany), GM12878 and GM11832 lymphoblastoid cell lines (LCL) were obtained through the Coriell Institute (Camden NJ, US). Cell lines were maintained at 37 °C, with 5% CO_2_ in either RPMI with 10% FBS (REH, GM12878 and GM11832), 20% FBS RMPI (MHH-CALL2) or 10% DMEM (HeLa) supplemented with GlutaMAX (ThermoFisher Scientific, Waltham, MA, USA). REH BCP-ALL cell line used as a model of *ETV6:RUNX1* translocation positive ALL. Cell line identity confirmed by translocation specific PCR primers available on request. HeLa cell line used as a non B-cell control. Cell lines were tested for mycoplasma (PCR Mycoplasma Test Kit I/C, PromoCell, Heidelberg, Germany), no positive results were obtained.

### Plasmid construction and luciferase assays

The putative regulatory regions containing rs7090445 and rs7896246 were amplified from genomic DNA from the CEU LCL cell lines NA12004 and NA10851 respectively using primers detailed in Supplementary Table 1. Gel purified PCR-products (Qiagen, Hilden, Germany) were A-tailed using 2U Thermprime DNA polymerase (ThermoFisher Scientific) and 200 μM dATP for 30 min at 70 °C, and cloned into pCR/8/GW/TOPO (ThermoFisher Scientific). Bacterial colonies were picked, cultured and purified using Qiagen Mini-prep Kit. SNP risk alleles were generated using site-directed mutagenesis (Quick Change XL, Agilent). Risk and non-risk variants were transferred into a Gateway compatible pGL3 Promoter vector using Gateway LR Clonase II (ThermoFisher Scientific) and pGL3 constructs purified using Qiagen Midi-prep kits. Inserts and single base changes were verified by Sanger sequencing. Site-directed mutagenesis and sequencing primers are detailed in Supplementary Tables 2 and 3. Cells were transiently transfected by electroporation using a Nucleofector 2B (Lonza, Basel, Switzerland). 4 × 10^6^ MHH-CALL2, 3 × 10^6^ GM11832 and 2 × 10^6^ SEM cells were transfected with 2.5 μg of pGL3 promoter construct and 50 ng of pRL-SV40 (Promega, Madison, WI, United States ), for normalization in 100 μl of Solution V using either program X-01 (MHH-CALL2 and GM11832) or T-02 (SEM). After 16 h promoter activity was assayed using the Dual-Luciferase reporter assay system (Promega) using a Fluoroskan Ascent FL (Labsystems). Six biological replicates each containing 3 technical replicates, were performed for MHH-CALL2 and SEM.

### ChIP-seq

Proteins binding rs7896246 and rs7090445 were inferred from Encode ChIP-Seq data (http://hgdownload.cse.ucsc.edu/goldenPath/hg19/encodeDCC/wgEncodeRegTfbsClustered/ wgEncodeRegTfbsClusteredV3). ChIP-seq fastq data for proteins binding to either loci was downloaded from Encode (http://hgdownload.cse.ucsc.edu/goldenPath/hg19/encodeDCC/) and whole genome fastq data for the GM12878 was downloaded from Illumina Platinum Genomes project (AC:ERR194147). Fastq data were aligned to the human reference genome (human_g1k_v37), edited to mask common SNPs in build 141, using Stampy v1.0.28 sequence alignment tool. Allele specificity of ChIP-seq data was assessed using a binomial test of allele specific read counts, assuming an equal distribution. Biological replicates were analysed separately and combined using Fisher’s method.

### Electromobility shift assay

Nuclear protein was extracted from GM12878 cells using NE-PER nuclear and cytoplasmic extraction kits (Thermo Fisher Scientific). Fluorescently labelled (DayLight 682 nM) and unlabelled complementary oligonucleotides (Eurofins Genomics) flanking rs7090445 (5′-AACAGCCTAACCTAG GTTAT[**T**/**C**]GATAGCTTTGAGACCTTCTG-3′) were annealed to generate double-stranded EMSA probes. Each 20 μl binding reaction contained 50 fmol labelled target DNA, 1 × binding buffer (10 mM Tris, 30 mM KCl, 2 mM DTT, 2.5% glycerol, 0.01 mg ml^−1^ BSA [pH 7.5], 1 μg polydI-dC [Sigma-Aldrich] and 10 μg nuclear protein extract). Reactions were incubated in the dark for 30 min at room temperature. Competition assays were performed by adding 200-fold molar excess of unlabelled probes. Super-shifts were performed by adding 2 μg RUNX3 antibody (Santa Cruz Biotechnology, sc-376591) to the binding reaction and incubating for 15 min before the addition of labelled probe. DNA-protein complexes were resolved by electrophoresis on a 6% DNA retardation gel (Life Technologies) in 0.5 × Tris-borate-EDTA (TBE) at 4 °C. Gels were imaged using the Odyssey Fc Infrared Imaging System (LI-COR Biosciences). An uncropped EMSA gel image is provided in Supplementary Fig. 9.

### ChIP q-RT PCR

ChIP was performed using the Active Motif ChIP-IT Express kit (Active Motif, La Hulpe Belgium), with the modification that after mircococcal nuclease digestion of DNA, cells were sonicated in a Biorupter for 10 min at 4 °C, to aid chromatin release. 25 μg of chromatin was incubated with 1 μg of anti-RUNX3 (Santa Cruz sc-376591) or 1 μg IgG2b (ThermoFisher Scientic, MA5-14447). After washing qRT-PCR was performed using primer sequences detailed in Supplementary Table 6. Three biological replicates were performed using separate chromatin preparations. Interpolated quantities for each target were normalized to an input DNA sample, without antibody pull-down.

### Hi-C data analysis

Hi-C data derived from GM12878, digested with MboI, was retrieved from the NCBI (http://www.ncbi.nlm.nih.gov/geo/query/acc.cgi?acc=GSE63525)^20^. Data from replicates were combined and analysed using a balanced Knight-Ruiz normalization method^34^, and viewed at 5 kb resolution.

### Chromatin confirmation capture

Chromatin confirmation capture (3C) libraries were generated according to Naumova *et al*.^35^ A control 3C template was generated using minimally overlapping BAC clones RPCI20I18 (RP11-20I18) and RPCI508P23 (RP11-508P23) (Source Bioscience, Cambridge UK). Taqman reactions were performed in triplicate with four biological replicates using 150 ng of DNA amplified using thermoprime DNA polymerase (ThermoFisher Scientific) and analysed using an ABI7900HT (Applied Biosystems). Raw data signals were normalized to ligated BAC clones and an internal loading control. Primer, probe sequences and cycling conditions are detailed in Supplementary Table 5. PCR-products were verified by Sanger sequencing.

### qRT-PCR

qRT-PCR was performed using SYBR Green PCR master mix (ThermoFisher Scientific). Samples were analysed undiluted for ChIP assays or 1/100 diluted in H_2_0 for siRNA assays. Each biological replicate contained three technical replicates. PCR quantitation was by standard curve method. Five-fold serial dilutions of DNA or complementary DNA (cDNA) were used to control for differential primer efficiency. For siRNA assays target gene expression was normalized to the geometric average of PPIA, TBP, G6PD and LAMIN A/C.

### siRNA transfection and reverse transcription

GM11832 homozygous for rs7090445 T-allele was transiently transfected by electroporation using 200 nM of siRNA (Eurofins Genomics, Ebersberg, Germany) for 48 h (siRNA sequences detailed in Supplementary Table 4) and then either re-transfected using the T-allele construct of pGL3 and pRL-SV40, or lysed. RNA was prepared using the Qiagen RNAeasy kit. 1–2 μg of RNA was reverse transcribed using MMLV (Promega) and 5 uM dT (ref. 14) per guidelines. Pooled cDNAs diluted 1/20 in H_2_0 were used for standard curve quantitation.

### Gene expression in BCP-ALL

mRNA expression data on paediatric B-cell ALL was retrieved from Gene Expression Omnibus accessions GSE13425 (*n*=154) and GSE13351 (*n*=92) and RMA-normalized using a custom array definition file from (http://brainarray.mbni.med.umich.edu/Brainarray/Database/CustomCDF/20.0.0/ensg.asp) using the R packages Affy and limma. RNA-seq data from 139 B-cell ALL was quantified using RSEM as described previously^22^. Spearman correlation coefficient and associated *P* values were calculated in R version 3.2.2.

### SNP array and RNA sequencing

90 cases of HD-ALL were genotyped using either Illumina Human 1M-duo Infinium BeadChip, HumanOmni1-Quad BeadChip or IlluminaOmni5M BeadChips^27^,^36^. 45 of the 90 ALL blasts were also RNA sequenced on a HiScanSQ (Illumina, San Diego CA, US) as previously described^22^. Gene Expression levels were estimated by using TCGA UNC V2 RNA-seq workflow and normalized RNA-Seq by Expectation Maximization (RSEM) values were then log_2_(*x*+1) transformed. To control for ploidy effects only cases with chromosome 10 trisomy for were considered for genotype expression correlations and chromosome duplication bias tests.

### Data availability

Existing GWAS data is available from the authors upon request^13^. Existing RNA sequencing in ALL blasts is available from the authors on request^22^. Existing genotyping data in HD-ALL blasts is available from the authors upon request^27^. Publically available data used in this study can be found as deposited in the following data sets; DNAse I hypersensitivity data from GM12878: NCBI Gene Expression Omnibus GSE32970, DNAse I hypersensitivity data ALL blasts: European Genome-phenome Archive EGAD00001002499, H3K27ac/H3K4me1/H3K4me3 and H2AZ ChIP sequencing data from GM12878: NCBI Gene Expression Omnibus GSE29611, H3K27ac/H3K4me1/H3K4me3 sequencing data ALL blasts: European Genome-phenome Archive EGAS00001000326, RUNX3 ChIP-seq data from GM12878: NCBI Gene Expression Omnibus GSE32465, GM12878 whole genome sequencing: Illumina Platinum Genomes project AC:ERR194147, ChromHMM in GM12878: NCBI Gene Expression Omnibus GSE38163. Microarray expression data in ALL blasts: NCBI Gene Expression Omnibus GSE13425 and GSE13351. The remaining data within the Article and Supplementary Information files are available from the authors upon request.

## Additional information

**How to cite this article**: Studd, J. B. *et al*. Genetic and regulatory mechanism of susceptibility to high-hyperdiploid acute lymphoblastic leukaemia at 10q21.2. *Nat. Commun.* **8**, 14616 doi: 10.1038/ncomms14616 (2017).

**Publisher’s note**: Springer Nature remains neutral with regard to jurisdictional claims in published maps and institutional affiliations.

## Supplementary Material

Supplementary Information Supplementary Tables, Supplementary Figures, and Supplementary References
