# Author Correction: Genetic and regulatory mechanism of susceptibility to high-hyperdiploid acute lymphoblastic leukaemia at 10q21.2

**DOI:** 10.1038/ncomms16204

**Published:** 2018-05-25

**Authors:** James B. Studd, Jayaram Vijayakrishnan, Minjun Yang, Gabriele Migliorini, Kajsa Paulsson, Richard S. Houlston

Nature Communications 8: Article number: 14616; DOI: 10.1038/ncomms14616 (2017); Published online: 03 03 2017; Updated: 05 25 2018

The original version of this Article contained an error in the title, the final paragraph of the Introduction and the first subsection of the Results, where chromosome locus 10q21.2 was incorrectly referred to as 10p21.2. This has now been corrected in both the PDF and HTML versions of the Article.

