# Supplementary material for: Genetic and regulatory mechanism of susceptibility to high-hyperdiploid acute lymphoblastic leukaemia at 10q21.2
Source: Nat Commun. 2017 Mar 3;8:14616. doi: 10.1038/ncomms14616 (PMC5337971; doi:10.1038/ncomms14616)
Supplement: Supplementary Information — Supplementary Tables, Supplementary Figures, and Supplementary References [file ncomms14616-s1.pdf]

## Supplementary Material

### Supplementary Table 1: Luciferase assay primer sequences

| | | |
|---|---|---|
| rs7090445 | Forward 5'-3' | AATCCCAGGTGCTTATGGACA |
| rs7090445 | Reverse 5'-3' | TGAGCCGAGATTGCACCATT |
| rs7896246 | Forward 5'-3' | GTGTCCAGGAACTCCCAAGG |
| rs7896246 | Reverse 5'-3' | ATTCCGAATTCACACGTACAATTA |

### Supplementary Table 2: Site directed mutagenesis primers

| | | |
|---|---|---|
| rs7090445 | Forward 5'-3' | GAAGGTCTCAAAGCTATCGATAACCTAGGTTAGGCTGTTG |
| rs7090445 | Reverse 5'-3' | CAACAGCCTAACCTAGGTTATCGATAGCTTTGAGACCTTC |
| rs7896246 | Forward 5'-3' | AAACAAGGTGTTTGATTGGGGGAGATTTTCTTCTCAGAGT |
| rs7896246 | Reverse 5'-3' | ACTCTGAGAAGAAAATCTCCCCCAATCAAACACCTTGTTT |

### Supplementary Table 3: Sequencing primers

| | | |
|---|---|---|
| rs7090445 | Forward 5'-3' | TGACTTGGGTACCGCTAGTGTAT |
| rs7090445 | Reverse 5'-3' | AAGGGGCTAGGTATCAAACCA |

### Supplementary Table 4: siRNA sequences

| | | |
|---|---|---|
| RUNX3 #1 | Sense 5'-3' | GGAGAGAACAGGAGGGCCAUU-dTdT |
| RUNX3 #1 | Anti-sense 5'-3' | UGGCCCUCCUGUUCUCUCC-dAdC |
| RUNX3 #2 | Sense 5'-3' | GGCAGAAGCUGGAGGACCAUU-dTdT |
| RUNX3 #2 | Anti-sense 5'-3' | UGGUCCUCCAGCUUCUGCC-dGdG |
| RUNX3 #3 | Sense 5'-3' | GGAUGGAGCUGGGUGGAAA-dTdT |
| RUNX3 #3 | Anti-sense 5'-3' | UUUCCACCCAGCUCCAUCC-dTdC |
| Non Targeting | Sense 5'-3' | UGGUUUACAUGUCGACUAA-dTdT |
| Non Targeting | Anti-sense 5'-3' | UUAGUCGACAUGUAAACCA-dTdT |

### Supplementary Table 5: 3C primers and probe sequences

| Primer name | Base pair pos (hg19) | Distance from SNP | Sequence 5'-3' |
|---|---|---|---|
| Fragment 35 | 63,619,507 | 101,919 | GCAGGCTATAAAATGAAATAGCGGTATGATCCCA |
| Fragment 65 | 63,637,444 | 83,982 | GCCAGCTGATGGTGTGGAGTCTAACCTTTT |
| Fragment 79 | 63,646,148 | 75,278 | AGTAGAGCCCTGGAGGTAGTAGCACAGTTG |
| *Fragment 104 TSS | 63,660,099 | 61,327 | CCAGGCTAGATTGCATTTTCGCAGTGTTGA |

| | | | |
|---|---|---|---|
| Fragment 119 | 63,667,995 | 53,431 | TTTTGCATCTGTCTCTTCACCTAGCAGAGT |
| Fragment 129 | 63,675,439 | 45,987 | GCCTTAGTTTGCCTTAGCTTGCCGTGTTTT |
| Fragment 155 | 63,689,832 | 31,594 | CATTCTCTATGGGGATTCCAGTGCTCACCC |
| SNP | 63,721,221 | 205 | CCGGTTCCAACCCTTTCTAGCTTGTT |
| ~Probe | 63,721,180 | 41 | AGCTTTGAGACCTTCTGAGACCATGCAAGCCTCGTGT |

* Fragment 104 primer located proximal to the transcription start site of ARID5B

~ Probe synthesised by IDT full annotation

5' - /56-FAM/AGCTTTGAG/ZEN/ACCTTCTGAGACCATGCAAGCCTCGTGT/3IABkFQ/-3'

3C loading controls F – CTTTGCCGTGAAGCCAGCGC R – CGAAGGTATGTGGAGGGTAGGCA

3C cycling conditions; $95^0$C – 1 min; 40 cycles of – ($95^0$C – 10 sec, $66^0$C – 1 min)

**Supplementary Table 6: Quantitative real time PCR primers**

| | | |
|---|---|---|
| RUNX3 | Forward 5'-3' | AGGCAATGACGAGAACTACTCC |
| RUNX3 | Reverse 5'-3' | CGAAGGTCGTTGAACCTGG |
| ARID5B | Forward 5'-3' | TCTTAAAGGCAGACCACGCAA |
| ARID5B | Reverse 5'-3' | TGCCATCGGAATTGTTGTTGG |
| PPIA | Forward 5'-3' | CTGCACTGCCAAGACTGA |
| PPIA | Reverse 5'-3' | GCCATTCCTGGACCCAAA |
| TBP | Forward 5'-3' | TGCACAGGAGCCAAGAGTGAA |
| TBP | Reverse 5'-3' | CACATCACAGCTCCCCACCA |
| G6PD | Forward 5'-3' | GATGCCTTCCATCAGTCGGA |
| G6PD | Reverse 5'-3' | GCTCACTCTGTTTGCGGATG |
| LAMIN A/C | Forward 5'-3' | AGCAGCGTGAGTTTGAGAGC |
| LAMIN A/C | Reverse 5'-3' | AGACTGCCTGGCATTGTCC |
| ChIP rs7090445 | Forward 5'-3' | TTTCCTTTGAGGCTTTTCTCTTGG |
| ChIP rs7090445 | Reverse 5'-3' | TTGACACGAGGCTTGCATGG |

Q-RT-PCR cycling conditions;

$95^0$C – 10 min; 40 cycles of - ($95^0$C – 10 sec; $58^0$C – 20 sec)

**Supplementary Table 7: Genotyping of the lead SNPs in 10q21.**

| | | UK GWAS | | German GWAS | |
|---|---|---|---|---|---|
| **rsID** | **Chr10 pos (hg19)** | **Cases** | **Controls** | **Cases** | **Controls** |
| rs10821936 | 63,723,577 | Imputed | Imputed | Imputed | Imputed |
| rs4245595 | 63,722,895 | Imputed | Imputed | Imputed | Imputed |
| rs7090445 | 63,721,176 | Imputed | Imputed | Typed | Typed |
| *rs10821937* | 63,723,909 | Imputed | Imputed | Imputed | Imputed |
| rs7896246 | 63,724,390 | Imputed | Imputed | Imputed | Imputed |
| rs4948492 | 63,719,739 | Imputed | Imputed | Imputed | Imputed |
| rs4245597 | 63,725,942 | Imputed | Imputed | Imputed | Imputed |
| rs4506592 | 63,727,187 | Imputed | Imputed | Typed | Typed |

Method of genotype assessment for lead SNPs in the 10q21 ALL risk locus.

**Supplementary Table 8: Lead SNPs at the chromosome 10q21 risk locus.**

| rsID | Chr10 pos (hg19) | Risk Allele | Non-Risk Allele | MAF | *P*-value | | Odds Ratio | | |
|---|---|---|---|---|---|---|---|---|---|
| | | | | | B-ALL | HD- ALL | B - ALL | HD - ALL | $R^2$ |
| rs10821936 | 63,723,577 | C | T | 0.36 | $5.13x10^{-50}$ | $7.12x10^{-39}$ | 1.87 | 2.57 | 1.00 |
| rs4245595 | 63,722,895 | C | T | 0.36 | $1.13x10^{-49}$ | $1.06x11^{-38}$ | 1.87 | 2.57 | 1.00 |
| rs7090445 | 63,721,176 | C | T | 0.36 | $1.15x10^{-49}$ | $1.53x12^{-38}$ | 1.87 | 2.55 | 0.98 |
| rs10821937 | 63,723,909 | C | T | 0.36 | $1.33x10^{-49}$ | $1.47x13^{-38}$ | 1.87 | 2.55 | 1.00 |
| rs7896246 | 63,724,390 | A | G | 0.36 | $2.51x10^{-49}$ | $1.36x14^{-38}$ | 1.86 | 2.56 | 1.00 |
| rs4948492 | 63,719,739 | C | T | 0.36 | $2.89x10^{-49}$ | $1.42x15^{-38}$ | 1.87 | 2.56 | 0.98 |
| rs4245597 | 63,725,942 | G | A | 0.36 | $5.32x10^{-49}$ | $2.18x16^{-38}$ | 1.86 | 2.55 | 0.98 |
| rs4506592 | 63,727,187 | A | G | 0.38 | $1.42x10^{-44}$ | $1.58x17^{-36}$ | 1.79 | 2.46 | 0.88 |

Lead SNPs in the 10q21 ALL risk locus displayed in *P*-value order (fixed effects meta-analysis of logistic regression *P*-value). MAF (minor allele frequency). $R^2$ linkage disequilibrium to lead SNP rs10821936 calculated from UK10K and European 1000 Genome individuals.

**Supplementary Table 9**: **Functional annotation of lead SNPs in 10q21.**

| SNP rsID | ChIP-seq B-cell | Predicted motif Disruption | PhastCons | GERP |
|---|---|---|---|---|
| rs4948492 | | RXRA, SOX1, SOX13, EP300 | 0.011 | 1.65 |
| rs7090445 | POLR2A, EP300, ATF2, FOXM1, MEF2A, MEF2C, NFIC, RUNX3, RELA | CUX1, HOXD8, MEF2A | 0.007 | 0.274 |
| rs4245595 | | FOXJ2, MYEF2, MEIS1, MYC | 0 | 2.71 |
| rs10821936 | | CTCF, RUNX1 | 0 | -0.986 |
| rs10821937 | EBF1 | | 0.004 | 1.91 |
| rs7896246 | FOXM1, MEF2A | NR1H4, RREB1, | 1 | 4.11 |
| rs4245597 | ATF2 | DMRT1, HDX, POU1F1, SOX1, FOXN1, EP300 | 0.148 | 1.96 |
| rs4506592 | | CCNT2, EWSR1-FLI1, GATA1, HDAC2, TAL1 | 0.001 | -1.91 |

Tabular view and data values used in compiling Figure 1. Source data used to compile Figure 1 and Supplementary Figure 1: PhastCons - phastConsElements46way.txt.gz; GERP - allHg19RS_BW.txt.gz;
ChIP-seq - wgEncodeRegTfbsClusteredWithCellsV3.txt.gz (only transcription factor binding events in B-cell lines are shown. Predicted motif disruption extracted from HaploReg v4.1 (http://archive.broadinstitute.org/mammals/haploreg/haploreg.php).

**Supplementary Table 10: Functional annotation of lead SNPs in 10q21, histone modifications.**

| | Lymphoblastoid cell line GM12878 | | | | | ALL bast sample S017E3 | | | | ALL bast sample S017GSD | | | |
|---|---|---|---|---|---|---|---|---|---|---|---|---|---|
| | DNAse I HS | H3K4 me1 | H3K4 me3 | H3K27 ac | H2AZ | DNAse I HS | H3K4 me1 | H3K4 me3 | H3K27 ac | DNAse I HS | H3K4 me1 | H3K4 me3 | H3K27 ac |
| rs4948492 | 0 | 17.64 | 10 | 16.32 | 16.32 | 0 | 6.3 | 0.7 | 5.1 | 0 | 4.7 | 3.8 | 2.7 |
| **rs7090445** | 25 | 9.8 | 19.6 | 23.1 | 23.2 | 27.3 | 5.6 | 5.6 | 8.7 | 17.0 | 3.8 | 2.6 | 7.3 |
| rs4245595 | 0 | 17.4 | 7.0 | 15.6 | 15.6 | 0 | 5.6 | 0 | 4.8 | 0 | 2.3 | 0 | 2 |
| *rs10821936* | 0 | 41.2 | 20.1 | 24.2 | 24.2 | 0 | 5.8 | 0 | 8.4 | 0 | 1.4 | 0.7 | 3.7 |
| rs10821937 | 0 | 31.2 | 25.2 | 23.7 | 23.7 | 0 | 7.4 | 0 | 11.4 | 0 | 4.6 | 0 | 2.3 |
| **rs7896246** | 0 | 17.1 | 12.6 | 11.2 | 11.2 | 6.3 | 8.8 | 2.6 | 5 | 0 | 2.9 | 3.9 | 0 |
| rs4245597 | 0 | 16.6 | 19.9 | 11.4 | 11.4 | 7.5 | 2.9 | 0 | 4.4 | 4.7 | 3.5 | 0 | 2.7 |
| rs4506592 | 0 | 5.5 | 2 | 3 | 3 | 0 | 0.8 | 0.9 | 2.6 | 0 | 1.8 | 1.3 | 0 |

Numerical data values used in compiling Figure1

Source data used to compile Figure 1 and Supplementary Table 9: GM12878 data downloaded from http://hgdownload.cse.ucsc.edu/goldenPath/hg19/encodeDCC/.
ALL bast data downloaded from ftp://ftp.ebi.ac.uk/pub/databases/blueprint/data/homo_sapiens/GRCh38/bone_marrow/. **H2AZ** - wgEncodeBroadHistoneGm12878H2azStdPk.txt.gz. H3K27ac - wgEncodeBroadHistoneGm12878H3k27acStdPk.txt.gz, S017E3H1.ERX1345474.H3K27ac.bwa.GRCh38.20160309.bw, S01GSDH1.ERX1304206.H3K27ac.bwa.GRCh38.20160226. **H3H4me1** - wgEncodeBroadHistoneGm12878H3k4me1StdPk.txt.gz, S017E3H1.ERX1305324.H3K4me1.bwa.GRCh38.20160226.bw, S01GSDH1.ERX1347919.H3K4me1.bwa.GRCh38.20160309. **H3K4me3** - wgEncodeBroadHistoneGm12878H3k4me3StdPk.txt.gz, S017E3H1.ERX1345473.H3K4me3.bwa.GRCh38.20160309.bw, S01GSDH1.ERX1304205.H3K4me3.bwa.GRCh38.20160226. **DNAse I HS** - wgEncodeOpenChromDnaseGm12878Pk.txt.gz, S00GNV41.ERX1139714.Dnase.GRCh38.hotspot.20151104.bb, S00DFM41.ERX1139711.Dnase.GRCh38.hotspot.20151104.bb

**Supplementary Table 11: Cell line genotype for rs7090445**

| Cell line | rs7090445 genotype |
|---|---|
| **GM12878** | C\|T |
| **GM11832** | T\|T |
| **MHH-CALL2** | C\|C |
| **REH** | C\|T |
| **HeLa** | T\|T |

Genotypes established using Sanger sequencing.

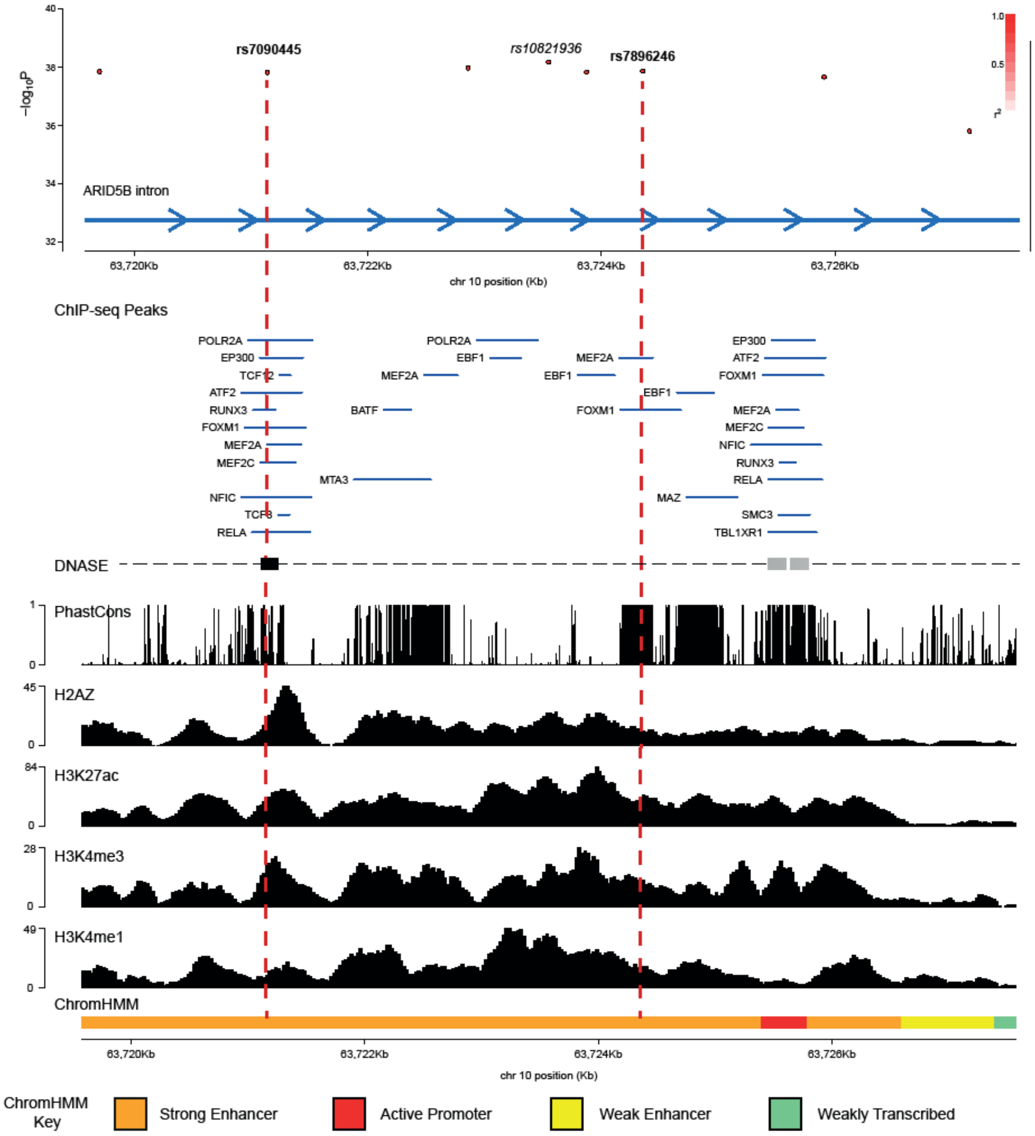


**Supplementary Figure 1: Genetic mapping and epigenetic landscape of the 10q21 HD-ALL risk locus, 8Kb view.** The HD-ALL risk region maps to a 8kb haplotype block within intron 3 of *ARID5B*. SNPs are shown as dots based on their chromosomal position (GRCh37/hg19 human genome build) on the x axis and -$\log^{10}$ association *P*-value in HD-ALL on the y axis. Colour intensity of each SNP reflects the extent of linkage disequilibrium (white $r^2$ = 0 to dark red $r^2$ = 1) with the lead SNP, rs10821936 (shown in black). Candidate functional SNPs rs7090445 and rs7896246 are highlighted (red dotted lines). Transcription factor ChIP-seq peaks from GM12878 (blue bars) are shown in the middle panel, annotated with the ChIP'd TF name. Evolutionary conservation quantified by PhastCons score, shown below. ChIP-seq data for H3K4Me1, H3K4Me3, and H3K27Ac histone modifications, the histone variant H2AZ and DNaseI hypersensitivity (DNASe) in the lymphoblastoid cell line GM12878 from ENCODE Project, are shown in the lower panel. Chromatin state segmentation (ChromHMM) in GM12878 from ENCODE project data are shown with colour key.

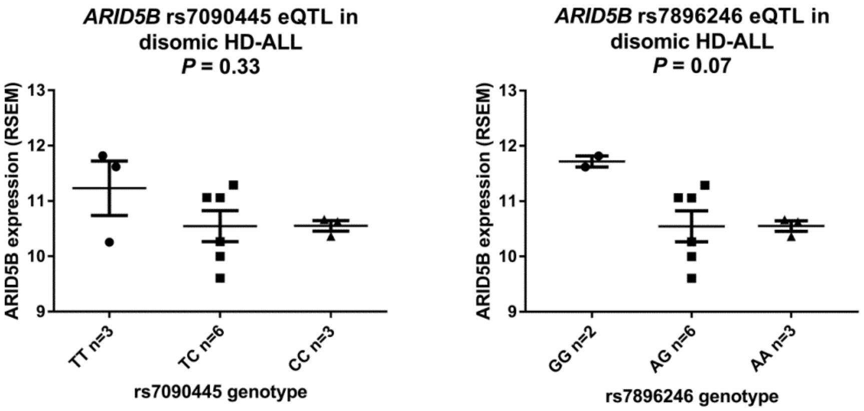


**Supplementary Figure 2: Risk alleles of rs7090445 and rs7896246 are associated with lower *ARID5B* expression.** Expression quantitative trait loci (eQTL) analysis performed in HD-ALL disomic for chromosome 10, plot shows mean and SEM of *ARID5B* expression. *P*-value calculated using ANOVA test.

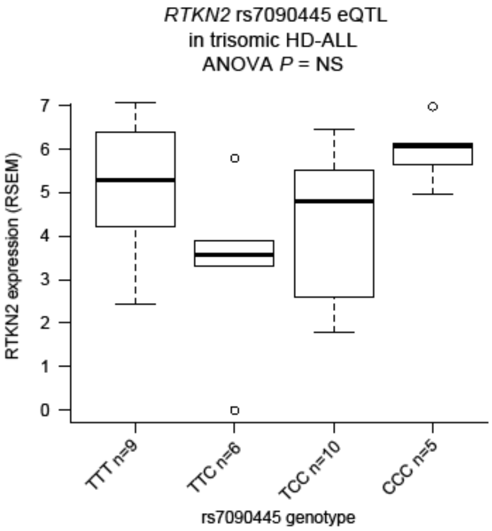


**Supplementary Figure 3: Risk allele of rs7090445 is not significantly associated with *RTKN2* expression.** Expression quantitative trait loci (eQTL) analysis performed in HD-ALL trisomic for chromosome 10.

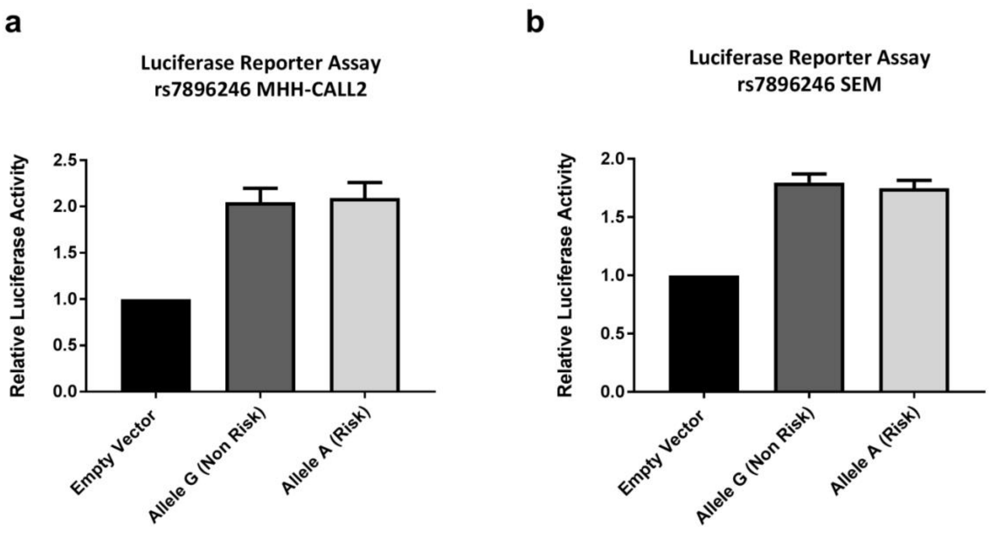


**Supplementary Figure 4: Risk allele of rs7896246 has no effect on enhancer activity.** Allele-specific constructs containing the 664bp putative regulatory sequence flanking rs7896246 were cloned into the pGL3-promoter luciferase reporter vector. The ratio of luminescence from the experimental pGL3-rs7896246 constructs to the Renilla internal control, pRL-SV40, was normalized to the empty pGL3-SV40 promoter vector. Data shown are mean ± SEM from 6 independent experiments performed in triplicate. The rs7896246 risk allele has no effect on enhancer activity in (**a**) MHH-CALL2 or (**b**) SEM cell lines.

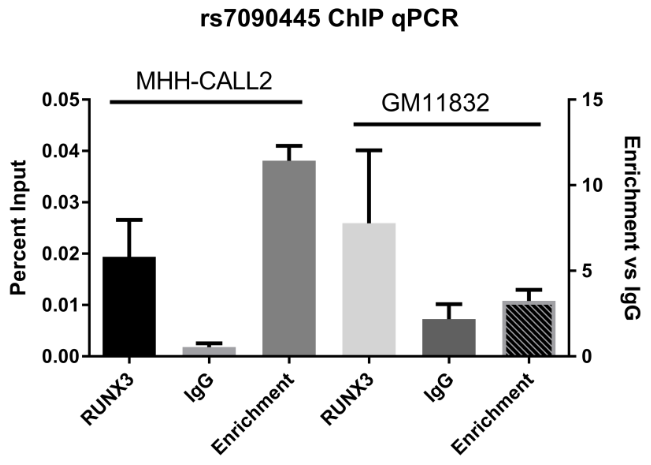


**Supplementary Figure 5: ChIP-qPCR for sequence flanking rs7090445 in MHH-CALL2 and GM11832.** Chromatin was pulled down with either anti-RUNX3 or IgG isotype control and amplified with primers surrounding rs7090445, data shown relative to input DNA, (n=3). Enrichment columns show the fold enrichment relative to IgG isotype controls, scale on right y axis.

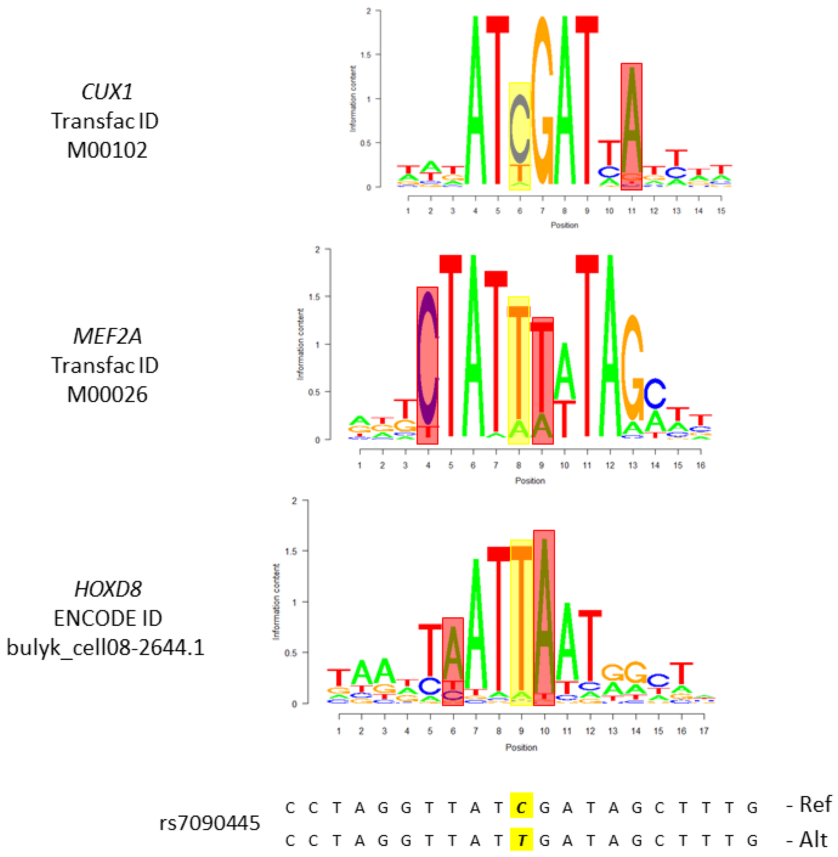


**Supplementary Figure 6: Position weight matrices (PWM) for motifs predicted to be disrupted by rs7090445.** Predicted disrupted motifs were extracted from HalpoReg v4.1[1]. PWMs for *CUX1*, *MEF2A* and *HOXD8* are aligned to sequence flanking rs7090445 highlighted in yellow. Significant mismatches are shown in red.

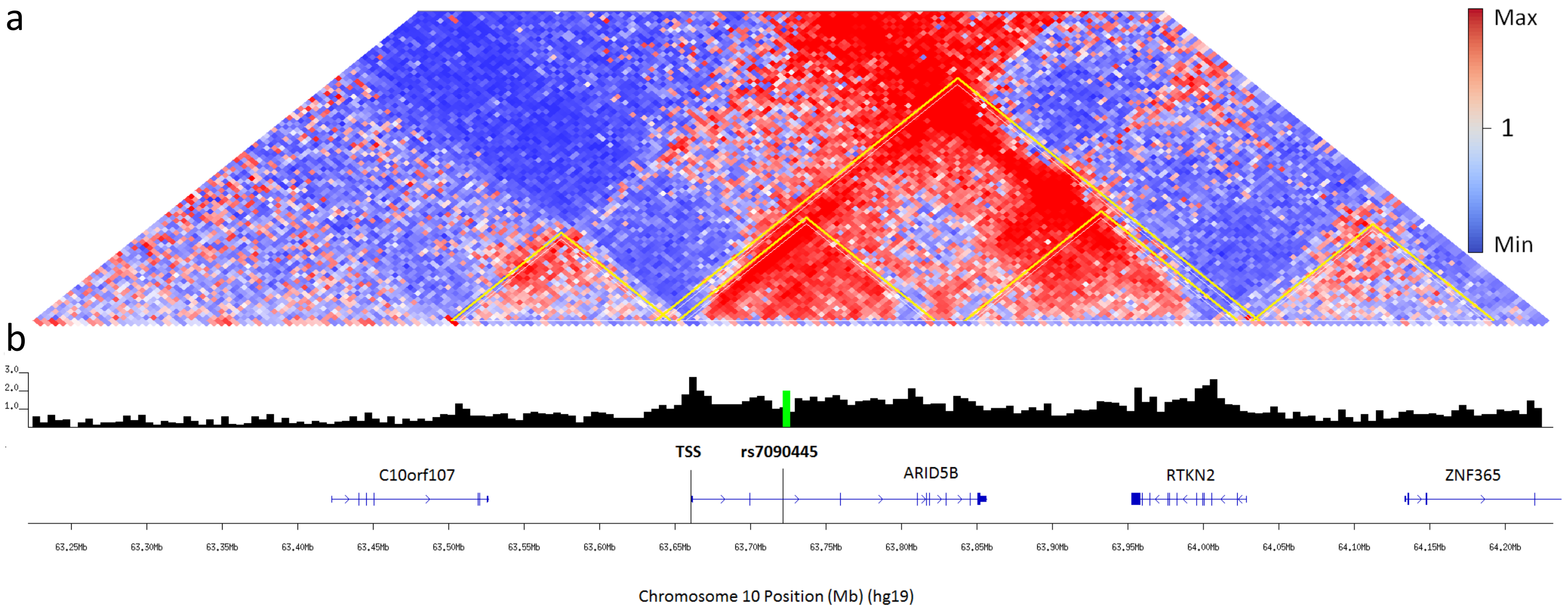


**Supplementary Figure 7: The transcription start site (TSS) of *ARID5B* and rs7090445 physically interact.** (**A**) HiC heat map, at 5kb resolution, based on data from Rao *et al*[2]. Enriched interaction frequencies are shown in red and depleted interactions in blue. Data from combined MboI experiments in GM12878, shown using Knight-Ruiz normalised observed / expected interaction frequencies. Significant TADs, as called in Rao et al[2] are shown in yellow. (**B**) 4C like plot showing pairwise interactions frequencies for each 5kb bin with the rs7090445 risk region.

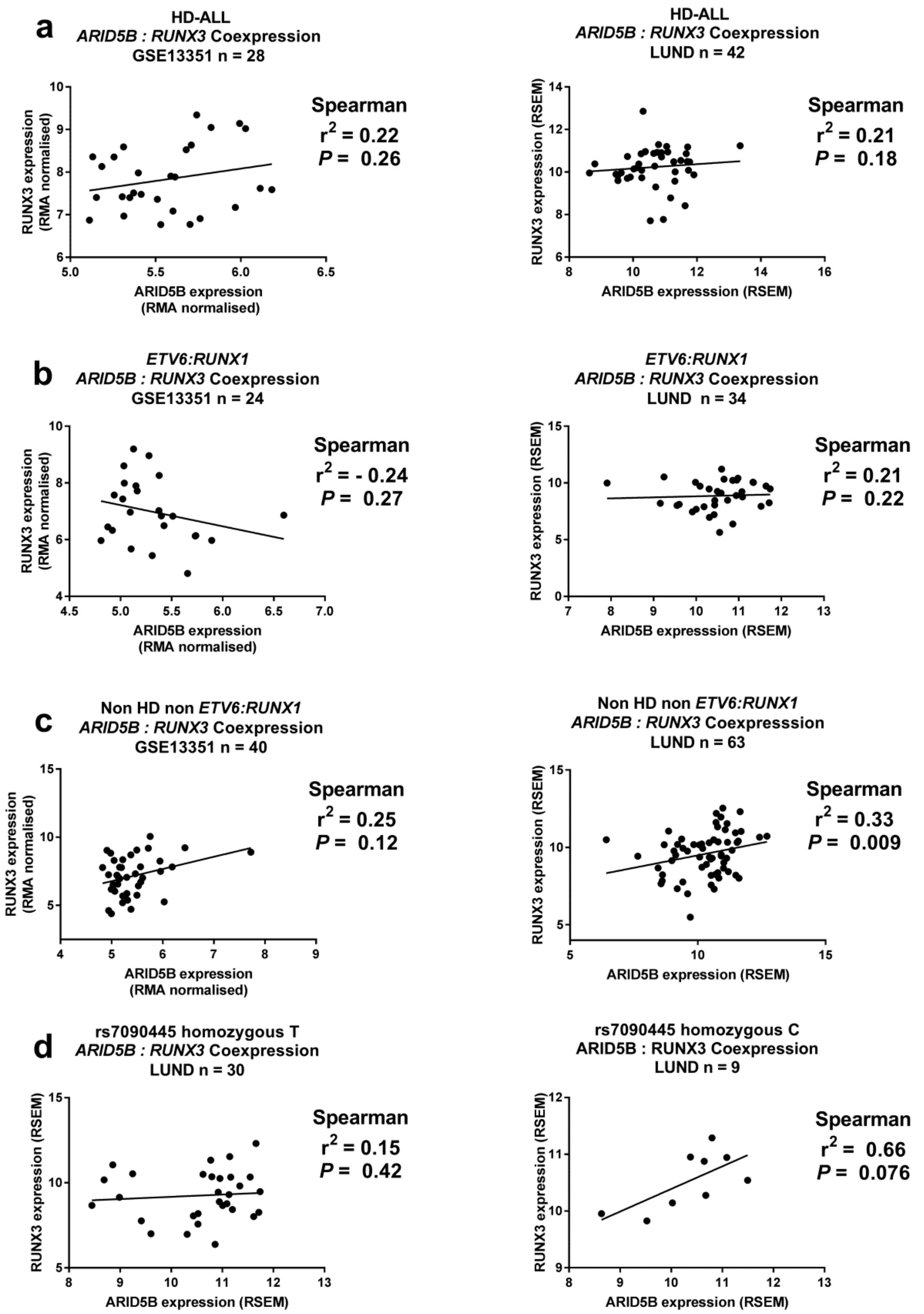


**Supplementary Figure 8:** Expression correlation between *ARID5B* and *RUNX3* in (**a**) hyperdiploid ALL (**b**) *ETV6:RUNX1* ALL and (**c**) non hyperdiploid non ETV6:RUNX1 ALL cases in the GSE13351 and LUND [3] datasets. (**d**) Expression correlation between *ARID5B* and *RUNX3* subdivided by genotype for rs7090445 in the LUND dataset.

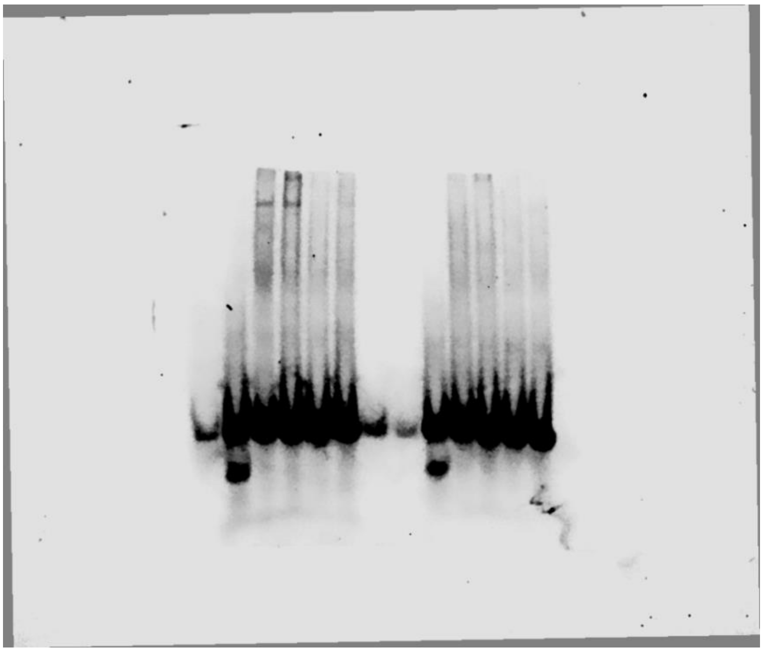

**Supplementary Figure 9:** Electromobility shift assay (EMSA) in GM12878. An uncropped original EMSA gel image of that used in **Fig 3.**

## References

1. Ward, L. D. & Kellis, M. HaploReg: A resource for exploring chromatin states, conservation, and regulatory motif alterations within sets of genetically linked variants. *Nucleic Acids Res.* **40,** D930-4 (2012).

2. Rao, S. S. P. *et al.* A 3D map of the human genome at kilobase resolution reveals principles of chromatin looping. *Cell* **159,** 1665–1680 (2014).

3. Lilljebjörn, H. *et al.* Identification of ETV6-RUNX1-like and DUX4-rearranged subtypes in paediatric B-cell precursor acute lymphoblastic leukaemia. *Nat. Commun.* **7,** 11790 (2016).
